# Parenting and parenting resources among Chinese parents with children under three years of age: rural and urban differences

**DOI:** 10.1186/s12875-023-01993-y

**Published:** 2023-02-01

**Authors:** Jing Han, Yinjun Hao, Naixue Cui, Zhenhui Wang, Pingping Lyu, Lei Yue

**Affiliations:** 1 School of Nursing and Rehabilitation, Cheeloo College of Medicine, Shandong University, No. 44 Wenhua Xi Road, Jinan, 250012 Shandong China; 2 Department of Healthcare Insurance, Beijing Hospital, No. 1 Dongdandahua Road, Dongcheng District, Beijing, 100730 China

**Keywords:** Rural–urban differences, Parenting, Parenting resources, Early childhood

## Abstract

**Background:**

Parenting is essential for children’s development and preventing child abuse and neglect. Providing parenting services within the primary health care settings demonstrated effectiveness in improving parenting quality. However, little is known about the status of parenting and parenting resources in rural areas and whether they differ between rural and urban areas in Mainland China.

**Objective:**

This study aimed to examine the rural–urban differences in parenting and availability of, utilization of, and need for parenting resources among Chinese parents with children under three years of age.

**Participants and setting:**

A total of 425 parents of children under three years of age participated in an online survey between March and May 2020.

**Methods:**

The Parenting and Family Adjustment Scale and Child Adjustment and Parenting Efficacy Scale were used to assess parenting, family adjustment, and parenting efficacy. The availability of, utilization of, and need for parenting resources were measured using self-developed questions based on literature. Chi-square tests,* t* tests, and Wilcoxon rank-sum test were used to examine the differences in responses between parents in rural and urban areas.

**Results:**

Compared with their urban counterparts, rural parents reported a higher level of negative parenting and more limited parenting resources. Both rural and urban parents reported low availability and utilization of parenting resources as well as a great need for parenting support services.

**Conclusions:**

Rural parents faced more parenting challenges and limited parenting resources compared with urban parents. Both rural and urban parents with children under three years of age reported great needs for parenting resources. These findings highlight the potential of delivering accessible, sustainable, and cost-effective parenting programs via the primary health care system for public welfare in both urban and rural areas, with more attention paid to rural parents to help them improve their parenting.

**Supplementary Information:**

The online version contains supplementary material available at 10.1186/s12875-023-01993-y.

## Introduction

Infancy and toddlerhood are important periods of individual growth and development, during which parenting plays a key role [1–4]. Previous studies have extensively examined the association between parenting in early life and children’s developmental outcomes and found that positive parenting (indicated by high levels of positive control, sensitivity, and warmth) was conducive to better physical, mental, and behavioral health outcomes, whereas negative parenting (indicated by high levels of negative control, hostility, and rejection) was related to poor developmental outcomes [5–7]. Negative parenting behaviors, such as overreaction and hostility are also risk factors for child abuse and neglect [8–10].

Recognizing the importance of high-quality parenting in early childhood, various parenting programs or services are provided to support families in child-rearing, such as Head Start, Sure Start, the Positive Parenting Program (Triple P), and many others. These programs show effectiveness in alleviating parental stress, enhancing parenting skills, promoting child development (e.g., language, motor, cognition, etc.), and preventing child abuse and neglect [11–14]. However, there is a social gradient in the awareness and use of services, so that at-risk families are particularly difficult to reach through universal services, and regional disparities exacerbate this problem. It is suggested that providing parenting services within the primary health care settings can make health services family-centered and easily accessible [15] and mitigate the effect of poverty-related disparity on child development as the primary health care setting is uniquely positioned to access at-risk populations and universally deliver preventive interventions at relatively low cost [16]. Studies reported that parenting programs (e.g., parenting education, consulting) provided by primary health care practitioners, such as home visiting nurses, child developmental specialists demonstrated positive effects on parenting behaviors [17, 18], parenting stress [19], parent–child interactions [20], child cognitive and language development [17, 18, 21] and child behavioral problems [22].

However, despite the many efforts to improve positive parenting, the disparity in parenting and parenting resources between rural and urban areas is common. Many studies across cultural contexts reported that, compared to their urban counterparts, rural parents tended to adopt an over-controlling [23] and intrusive [24] parenting style and to accept physical and emotional abuse as a legitimate strategy to discipline children [25]. The disparities in parenting styles and practices may be worsened by the fact that rural parents usually have fewer resources to support child-rearing. Prior studies suggested that it might be uniquely difficult for rural families to develop and maintain positive parenting practices because of the lack of support systems and amenities [26–28]. The rural–urban disparities of parenting style and resources are also evident in the Chinese context. Existing studies reported that rural parents with children of different ages were more likely to adopt a negative parenting style, whereas urban parents adopted a positive one [29, 30]. Researchers also found that Chinese rural parents tended to scold or hit their children when children behaved badly [31, 32].

China is adopting the “family doctor” approach to promote primary health care. The family doctor is responsible for basic medical care, public health, and contracted health management services. A previous study showed that 38.6% residents in rural areas and 35.7% in urban areas signed with family doctors in 2019 [33], and the rate increased to 75.5% for all residents in 2020. The implementation of the “family doctor” may provide a precious opportunity to provide high-quality parenting services with population coverage, especially in rural areas. A better understanding of the rural and urban differences in parenting resources, accessibility, and needs can provide support for the increasing investment of parenting training programs, and facilitate the integration of parenting training programs and primary health care.

Therefore, this study aimed to (1) describe parenting, including parenting style, family adjustment, parenting self-efficacy, and parenting resources regarding its availability, utilization, and need; and (2) examine the rural–urban disparities in parenting and parenting resources in a sample of Chinese parents with children under three years of age. Parenting style, family adjustment, and parenting self-efficacy were chosen as variables of interest because they were proposed as critical outcomes of both public health and individual or group parenting interventions [34, 35]. Parenting style was defined as a constellation of attitudes toward the child that are communicated to the child and create a specific emotional climate, family adjustment referred to parental emotional adjustment and partner and family support in parenting, and parenting self-efficacy was a parent's self-perception of their abilities to take care of their children, that is, the degree of confidence in their own parenting abilities [34, 35].

For this study, we operationally defined utilization of parenting services as the use of parenting services provided by private or public sectors for the purpose of obtaining information about parenting, promoting parenting skills and practice, and improving parent and child outcomes. According to the concept of access by Penchansky and Thomas [36, 37], utilization of health care is influenced by affordability, availability, accessibility, accommodation, and acceptability, and the five characteristics form a chain that is no stronger than its weakest. We focused on the availability of parenting services because limited availability of health care service was found to be an important barrier that reduces access to health services and increase risk of poor health outcomes [38–41], and the discrepancy of health care resources between rural and urban areas is evident. Availability of parenting services was operationally defined as parental awareness of requisite parenting resources that can meet their needs.

Previous studies [42–45] pointed out that population sociodemographic characteristics were demonstratively different by residence of location and can be mediators underlying the observed rural–urban discrepancies in many health outcomes. For example, Henning-Smith et al. examined the mediating effects of a series of individual characteristics including educational attainment, age, sex, race/ethnicity, living arrangement/marital status as mechanisms underlying the rural and urban differences in six Medicare quality outcomes [42]. In China, there are also substantial urban–rural differences in sociodemographic characteristics (e.g., household income, marital status) at population level [46]. According to the social ecological model, macrosystem such as societal factors that help create a climate or maintain economic or social inequalities in society can influence factors at individual, relationship, and community levels [47, 48].Therefore, in the present study, the rural and urban differences were examined at both bivariate and multivariate levels. For the latter, a range of sociodemographic characteristics that were disproportionate distributed across location of residence were operationalized as potential mediators and adjusted to illustrate the direct effect of rural/urban differences on the parenting variables of interest by removing the potential indirect effect through these individual characteristics.

## Methods

### Participants and procedure

An online survey was conducted using an online questionnaire platform Wenjuanxing (https://www.wjx.cn/) during March and May 2020 when China was hit by the COVID-19 pandemic. The pandemic center Wuhan City declared lockdown on January 23, 2020, and after that other areas of the country took stringent measures to contain the COVID-19 pandemic, such as shutting down enterprises, social distance, imposing quarantine policies according to the local response levels. To our knowledge, areas that were not affected much by COVID-19 started resuming normal operations in February, 2020. For example, in Shandong Province, where most of the participating parents were from, the order of production and life was gradually restored since February 23, 2020 according to the government regulation [49]. Considering the possible variabilities in severity of COVID-19 pandemic and local policy, we chose the snowball sampling strategy to recruit eligible parents who lived with a child under three years old and were able to understand the survey questions and provide responses through the online survey platform. In addition, primary health care practitioners in village clinics, community healthcare centers, and primary health care units of hospitals in rural and urban areas were asked to help disseminate the survey link to their clients as they were responsible for the primary care (routine growth and developmental examination and vaccination) of all children under six years old in their area under administration. For parents with more than one child under the age of 3 years, the youngest child under three years of age was regarded as the target child. Parents were able to participate in a raffle upon completion of the questionnaire to win WeChat (an instant communication application with virtual wallet and payment functions) credit of 1 to 10 Chinese Yuan (0.15 to 1.5 US Dollars) to compensate for their time taking the survey. The study was performed in accordance with the Declaration of Helsinki and approved by the Ethics Committee of the School of Nursing Shandong University (2018-R-022). A total of 425 parents filled the survey questionnaire, of which 167 were from the rural area and 258 were from the urban area.

### Variables and measurement

#### Sociodemographic information

Information of parent gender, age, marital status, child age, monthly household income, number of household members, number of children, education, occupation, other caregivers involved in childcare, residency location were collected. Monthly income per capita was calculated by dividing the midpoint of each monthly household income category by the number of household members and was used in the analysis. Residency location in village, town, and county was operationally defined as rural, and residency location of city was operationally defined as urban according to Chinese standards [50].

The Seventh National Census of China in 2020 reported rural and urban differences in population distributions (e.g., percentage of citizens aged 25–35 years with college or higher degree: urban 27.1% VS rural 3.2%; families with only one child: urban 54.3% VS rural 38.7%; income per capita: urban 3,529 RMB VS rural 1,335 RMB; marriage rate: urban 70.9% VS rural 73.9%) [46]. In addition, previous evidence also indicated that family income [51], parental age [51, 52], marital status [53], number of children in the household [54, 55], child age [52, 56], and help from other caregivers [57] were associated with parenting styles. Therefore, differences in parental age, monthly income per capita (CNY), marital status, number of children, age of the target child, and whether they had other caregivers caring for the child between rural and urban parents were affected by rural and urban residency (and hence, they were not confounders [58]) and may explain the rural–urban differences in parenting and parenting service utilization and needs, and hence were operationalized as mediators in the study.

#### Parenting and family adjustment

The Parenting and Family Adjustment Scale (PAFAS) was used to assess parenting and family adjustment. The PAFAS was developed by Sanders et al. [35]. The PAFAS consists of a parenting subscale (18 items) and a family adjustment subscale (12 items). The parenting subscale encompasses four factors, namely, Parental consistency (5 items), Coercive parenting (5 items), Positive encouragement (3 items), and Parent–child relationship (5 items). The family adjustment subscale has three factors, including Parental adjustment (5 items), Family relationships (4 items), and Parental teamwork (3 items). Items are rated on a 4-point scale from 0 (not true of me at all) to 4 (true of me very much), and some items are reverse scored. For each of the two subscales, the items are summed and then averaged to obtain the overall average scores, with higher scores indicating higher levels of parenting or family dysfunction. Scores on the measures have demonstrated good psychometric properties in an Australian sample [35], including good construct and concurrent validity as well as satisfactory internal consistency (coefficients ranged from 0.70 to 0.87). The Chinese version of PAFAS was translated and validated by Guo [59] among parents with children aged 2 to 12 years old and showed good construct validity, concurrent validity, convergent validity, and reliability (H coefficient ranging from 0.50–0.78 for all the factors). In this study, the parenting subscale was applied to parents with children above two years of age and the internal consistency McDonald’s Omega (ω) ranged from 0.54 to 0.85. The relatively low internal consistency of the Parenting consistency factor was consistent with Guo’s report [59].

Because the items of the family adjustment subscale were not age-specific, we applied this measure to all participating parents. To establish the psychometric property of this subscale in parents with children under two years of age, we performed confirmatory factor analysis (CFA) and internal consistency reliability. The CFA showed good model of fit (*χ*^2^ = 52.68, *p* = 0.071, Root Mean Square Error of Approximation (RMSEA) = 0.04, Comparative Fit Index (CFI) = 0.98, Tucker-Lewis Index (TLI) = 0.98, Standardized Root Mean Square Residual (SRMR) = 0.05), indicating good construct validity. The McDonald’s ω coefficients for the three factors of the family adjustment subscale ranged from 0.69 to 0.70, indicating good internal consistency. The McDonald’s ω coefficients of the family adjustment subscale in the total sample ranged from 0.67 to 0.74.

#### Parenting self-efficacy

Parenting self-efficacy was measured using the self-efficacy subscale of the Child Adjustment and Parenting Efficacy Scale (CAPES). The CAPES was developed by Morawska et al. [60] and was a parent report measure including CAPES intensity subscale (27 items) and CAPES self-efficacy subscale (19 items). The CAPES self-efficacy subscale assesses parents’ confidence in managing children’s behavioral and emotional adjustment problems described by the 19 items that are also used in the CAPES intensity subscale on a 1 (“certain I cannot do it”) to 10 (“certain I can do it”) scale. CAPES was translated and validated into the Chinese language by Guo [61] and validated among Chinese parents with children ranging from 2 to 12 years old.

We asked all participating parents to fill in this questionnaire, and if their child was under two years of age, an extra instruction was added (i.e., *If your child is under 2 years old, they may have not demonstrated some of the following behaviors. In this case, imagine scenarios when your child exhibits these behaviors when he/she grows up, answer the items based on how confident you will be to manage these behaviors.*). Psychometric property tests regarding this modified self-efficacy subscale among parents with children under two years of age showed good reliability (Cronbach’s alpha = 0.961) and construct validity (model of fit derived from the confirmatory factor analysis: *χ*^2^ = 254.80, *p* < 0.001, RMSEA = 0.08, CFI = 0.95. TLI = 0.94, SRMR = 0.04). The Cronbach’s alpha of the subscale in the total sample was 0.96.

#### Availability, utilization, and needs of parenting support services

Availability of parenting support services (e.g., parenting resources by primary health care providers or public daycare centers, public lectures or materials about parenting, etc.) was asked with three options, “Yes”, “No”, and “Don’t know”. Those who answered “Yes” were further directed to a question regarding utilization (“Have you ever accessed to or utilized the parenting support services” with “Yes” and “No” options). One question was asked to assess parents’ need for parenting support services with three choices (“Yes”, “No”, and “Unsure”).

### Statistical analysis

Data were described using frequency and percentage for nominal variables, median, and quartiles for monthly income per capita due to its skew distribution, and mean and standard deviation for other numerical variables in the total sample and by rural and urban areas. Chi-square tests, *t* tests, and Wilcoxon sum rank test were used to compare the differences between rural and urban parents.

In order to further elucidate the direct effect of rural and urban differences on the outcomes of interest by removing the potential indirect effect through the sociodemographic characteristics of the participants, we ran models controlling for the operationalized mediators using general linear for parenting, family adjustment, and parenting efficacy, binary logistic regression for utilization of parenting support services, and multinomial logistic regression for needs of parenting support services. The results of these regression models were presented in the Additional file 1:  Appendix.

The significance level was set at 0.05 (two-sided). Data were analyzed with Stata 15 for Windows (StataCorp, TX) and SPSS27 (IBM Corp, NY).

## Results

### Characteristics of participating parents

Sample characteristics were summarized in Table 1. Most of the respondents were mothers (79.1%). The average age of parents was 31.83 ± 4.62 years old, 84.7% of participants had a college or higher level of education, and 90.7% of parents rated their economic status as ordinary or good in the neighborhood. Nearly all the participants were married. Most parents had one or two children, and the average age of the youngest child was 22.50 ± 13.54 months.

**Table 1:** Characteristics of parent respondents

	Total *n* (%)/ Mean ± SD/ Median (Q1, Q3)	Rural *N* = 167 *n* (%)/ Mean ± SD/ Median (Q1, Q3)	Urban *N* = 258 *n* (%)/ Mean ± SD/ Median (Q1, Q3)	*χ*^2^/*Z*^a^*/t*	*p*
Parent				6.00	0.014
Father	89 (20.9)	45 (26.9)	44 (17.1)		
Mother	336 (79.1)	122 (73.1)	214 (82.9)		
Parents’ age (years)	31.83 ± 4.62	30.65 ± 5.30	32.60 ± 3.95	-4.16	< 0.001
Education level				66.08	< 0.001
High school or lower	65 (15.3)	55 (32.9)	10 (3.9)		
College or higher	360 (84.7)	112 (67.1)	248 (96.1)		
Occupation				60.73	< 0.001
Full-time parent	31 (7.3)	24 (14.4)	7 (2.7)		
Low skilled job	46 (10.8)	30 (18.0)	16 (6.2)		
Civil servant	47 (11.1)	24 (14.4)	23 (8.9)		
Skilled professionals	272 (64.0)	71 (42.5)	201 (77.9)		
Other	29 (6.8)	18 (10.8)	11 (4.3)		
Monthly income per capita (CNY)	4166.67 (2500, 6250)	3125 (2500, 4375)	4375 (3500, 8333.33)	-7.16	< 0.001
Marital status				0.49	0.483
Married	415 (97.7)	162 (97.0)	253 (98.1)		
Other^b^	10 (2.3)	5 (3.0)	5 (1.9)		
Number of children				4.24	0.120
1	256 (60.2)	91 (54.5)	165 (64.0)		
2	162 (38.1)	72 (43.1)	90 (34.9)		
> = 3	7 (1.6)	4 (2.4)	3 (1.2)		
Age of the youngest child (months)	22.50 ± 13.54	23.47 ± 13.48	21.87 ± 13.57	1.19	0.236
Other caregivers caring for the child					
Spouse	281 (66.8)	108 (65.5)	173 (67.6)	0.20	0.652
Grandparents	318 (75.5)	116 (70.3)	202 (78.9)	4.02	0.045
Others	44(10.5)	16(9.7)	28(10.9)	0.17	0.685

*SD* standard deviation, *Q1* First quartile, *Q3* Third quartile

^a^Wilcoxon rank-sum test

^b^other included divorced, remarried, and widowed. 1 CNY ≈ 0.15 USD. Chi-square tests, *t* tests, and Wilcoxon sum rank test were used to compare the differences between rural and urban parents

As compared with their urban counterparts, rural parents were younger, reported lower education level, were more likely to engage in non-skilled professional work and had lower average monthly income. See Table 1.

Regarding other caregivers of the targeted child, almost 70.0% of parent respondents reported that both the father and the mother were involved in childcare. About three-quarters of parents reported that grandparents helped with child-rearing, and this proportion was higher among urban parents than in rural parents. See Table 1.

### Rural–urban differences in parenting, family adjustment, and parenting efficacy

Compared with urban parents, rural parents reported a higher level of coercive parenting, a lower level of positive encouragement, and poorer parent–child relationship. Rural parents also reported poorer family adjustment, family relationships, and parental teamwork, as well as less confidence in managing a child’s emotional and behavioral problems. See Table 2.

**Table 2:** Rural–urban differences in parenting, family adjustment, and parenting efficacy

	Total Mean ± SD	Rural Mean ± SD	Urban Mean ± SD	*t*	*p*
Parenting					
Parental consistency	1.10 ± 0.57	1.18 ± 0.61	1.05 ± 0.54	1.43	0.154
Coercive parenting	1.06 ± 0.62	1.29 ± 0.52	0.92 ± 0.63	4.16	< 0.001
Positive encouragement	2.17 ± 0.67	2.01 ± 0.67	2.27 ± 0.65	-2.61	0.010
Parent–child relationship	2.31 ± 0.63	2.12 ± .66	2.45 ± .57	-3.76	< 0.001
Family adjustment					
Parental adjustment	0.94 ± 0.57	1.10 ± 0.60	0.83 ± 0.52	4.94	< 0.001
Family relationships	0.68 ± 0.59	0.81 ± 0.64	0.59 ± 0.55	3.79	< 0.001
Parental teamwork	0.88 ± 0.62	0.98 ± 0.62	0.83 ± 0.61	2.42	0.016
Parenting efficacy	7.31 ± 1.71	7.06 ± 1.79	7.47 ± 1.64	-2.38	0.018

*t* tests were used to compare the differences between rural and urban parents

### Rural–urban differences in parenting support services: Availability, utilization, and needs

Less than 30.0% of parents reported having parenting support services available in the neighborhood. Compared with urban parents, rural parents reported a lower level of availability of parenting support services. Among the parents who had access to parenting support services, 65.1% reported never using them and about 12% more rural than urban parents reported using the services, though such difference was lack of statistical difference (Table 3). About 80.0% of parents reported a need for parenting support services.

**Table 3:** Rural–urban differences in availability of, utilization of, and needs for public parenting

	Total *n* (%)	Rural *n* (%)	Urban *n* (%)	*χ*^2^	*p*
Availability of parenting support services				6.43	0.040
Yes	109 (25.9)	45 (27.3)	64 (25.0)		
No	149 (35.4)	68 (41.2)	81 (31.6)		
Don’t know	163 (38.7)	52 (31.5)	111 (43.4)		
Utilization of parenting support services				1.83	0.176
Yes	38 (34.9)	19 (42.2)	19 (29.7)		
No	71 (65.1)	26 (57.8)	45 (70.3)		
Needs for parenting support services				3.00	0.223
Yes	341 (81.0)	128 (77.6)	213 (83.2)		
No	26 (6.2)	14 (8.5)	12 (4.7)		
Unsure	54 (12.8)	23 (13.9)	31 (12.1)		

### Rural–urban differences from the adjusted models

Differences in coercive parenting, positive encouragement, parent–child relationship, parental adjustment and family relationships between rural and urban parents remained statistically significant after including the sociodemographic characteristics in the regression models, whereas the significance of the rural–urban differences in parental teamwork and parenting efficacy disappeared after the adjustment (Table S1). The absence of significant rural–urban differences in the utilization (Table S2) and needs (Table S3) of parenting support services remained after the including the sociodemographic characteristics in the regression model.

## Discussion

As one of the few studies to describe and compare the differences in parenting and parenting resources among Chinese rural and urban parents with children under three years of age, this study found that compared with urban parents, rural parents demonstrated a higher level of coercive parenting, poorer parent–child relationship, and family adjustment as well as a lower level of positive encouragement and self-efficacy. In addition, parents reported low levels of availability and utilization of parenting resources, which was even lower in rural parents than urban parents. Both rural and urban parents expressed a great need for parenting support services.

As expected, we found that compared with urban parents, rural parents reported a higher level of coercive parenting, poorer parent–child relationship, and family adjustment as well as a lower level of positive encouragement and self-efficacy, which were consistent with previous reports [23, 24]. Although after controlling for parents’ sociodemographic characteristics, the statistically significant rural–urban differences in parental teamwork and parenting efficacy disappear, other parenting variables still existed between rural and urban parents. This suggests that rural–urban differences in parenting and family adjustment cannot be solely attributed to differences in sociodemographic distributions between rural and urban areas. Other factors, especially modifiable ones (e.g., cultural beliefs and norms of parenting, awareness of importance of parenting to child development) need to be further estimated to understand the causes of such disparity.

In regards to the availability of parenting resources, we found that less than 30% of parents reported availability of parenting support services and rural parents reported fewer resources. The low availability of parenting resources was consistent with a previous study, which found that parenting resources for children aged 0–3 years and their families were very limited in mainland China [4]. Family service system is a system formed in cooperation with parents and social forces including kindergartens, health care institutions, etc. under the leadership of government agencies to promote the development of family education. The lack of implement of a formal family service system, the insufficient financial input in public services for families with very young children [4], and the shortage of relevant professionals [62] are the main reasons for the limited resources and should be tackled. Unawareness of available resources may be another potential reason for reporting unavailability, which we could not distinguish using the current data. If this was true, efforts of disseminating available resources to all parents need to be taken.

It should be noted that among parents reporting available parenting resources, very few utilized them. The few utilization was consistent with the previous finding that even in Shanghai (one of the most developed, first-tier metropolitan cities in China), only 23% of families with children under three years of age had received parenting services [63]. A previous Chinese study [3] also found that parenting services provided by the government have many deficiencies, such as lack of publicity, low sense of responsibility, and discontinuity of services; parents were unclear about when and where to get the parenting services, what they could benefit from the parenting support services, and worried about the quality and cost of parenting service provided by the private sectors (e.g., early education centers, parenting consulting institutes). Furthermore, we found that about 12% more rural parents than urban parents reported using parenting support services when available. However, this difference did not differ significantly between rural and urban parents, which could be because of lack of statistical power resulting from the small number (*n* = 19) of people who have used parenting support services in both rural and urban areas.

In the same line, we found a high proportion of parents (about 80%) reported a need for parenting support services. Such needs can be met by incorporating parenting services into the primary health care system, especially the family doctor system in China to make it accessible, sustainable, and cost-effective. According to the latest statistics by the National Health Commission of the People's Republic of China, more than 80% of Chinese residents could reach a primary health care center within 15 min, and 75.5% has signed with family doctors [64]. In addition, Chinese parents showed a preference for receiving parenting services nearby [62]. Previous evidence also supported that parenting programs carried out at home [65] or in primary health care settings [15, 16] were effective in improving parenting quality at a relatively low cost [16].

### Limitations

There are some limitations to this study. First, the participating parents were recruited through an online survey, and their representativeness was limited. About 70% of the participating parents are mainly from the same province (Shandong Province, China). In addition, the survey was distributed electronically, which means that the parents had to have a smart phone or other method for internet access. This left parents who did have access to these resources or unable to use them out. These parents might be more likely reside in rural areas, at higher risk of negative parenting and had more limited access to parenting support resources. Therefore, leaving these parents out might underestimate the true rural and urban differences. Besides, the percentage of participating parents aged 25–35 years with college or higher degree in our study (urban 96.1% VS rural 67.1%), the proportion of only-child family (urban 64.0% VS rural 54.5%), and family income (urban 4,375 RMB VS rural 3,125 RMB) were higher than the national average reported in the Seventh National Census of China in 2020 [46]. Further studies using representative samples are needed to replicate the findings.

Second, although many Chinese grandparents participated in child-rearing, they were not targeted in this survey because very few could use smartphones or computers to access the online survey, especially in rural areas.

Third, this online survey was conducted during the COVID-19 pandemic but we did not directly examine the effect of COVID-19 on parents’ service availability and utilization. Although in the instruction sections of the questionnaire, we emphasized to choose the most appropriate option based on their usual experiences, parental behaviors and perceptions of resources might be influenced by the quarantine, business, and public services shutdown as well as concerns of COVID-19 cross-infection. Nonetheless, the study findings can serve as a good reference for studies conducted in the post-pandemic era. We call for more studies on the parenting and parenting resources conducted not amid the pandemic so that the possible effect of the pandemic can be estimated.

Fourth, considering that there were two clear steps in parents' accessing services–the first being awareness of available services and then, if available, some parents chose to utilize and the other did not. However, we could not perform the two-step nested logit model because the independent variables were not varying within individual’s choice of utilization. We called for future research to collect variables at the utilization level within each individual case (e.g., perceived norm of and attitudes toward both utilizing and not utilizing parenting services) to proceed this two-step nested logit model. Finally, villages, townships, counties, and cities are relatively clearly divided in China; however, the self-reported residency location information was still likely to suffer from report bias.

Despite these limitations, this study still provides important evidence of rural and urban disparities of parenting and parenting resources in China. Our findings suggest that actions should be taken to build parenting resources of high quality and affordability and make them accessible to both urban and rural parents to meet their needs. Especially, rural parents warrant more attention due to their more negative parenting style, poorer family adjustment, and lower parenting efficiency. Our findings also suggest that primary health care practitioners, especially those in the rural areas with the privilege to identify parents’ needs in child-rearing can be trained to provide professional parenting services to meet parents’ needs and increase the accessibility of parenting resources at low cost. Alternatively, digital parenting services or interventions may be beneficial to help families in remote rural areas due to its advantage of easy access; however, the affordability and acceptability of digital services in rural areas still need to be addressed.

## Conclusions

This study found that rural parents reported more negative parenting, poorer family adjustment, lower parenting efficiency, and more limited parenting resources compared to their urban counterparts. Moreover, the availability and utilization of parenting resources were low in both rural and urban parents, and both categories expressed a great need for public parenting resources. These findings highlight the importance of enhancing the investment of high-quality, accessible, and affordable parenting resources in both urban and rural areas, with more attention paid to rural parents to help them improve their parenting. Primary health care professionals, such as family doctors in China can take the lead in providing such resources, especially in the rural areas.

## Supplementary Information

**Additional file 1: Table S1.** General linear regression of rural-urban differences in parenting, family adjustment, and parenting efficacy. **Table S2.** Binary logistic regression of rural-urban differences on utilization of parenting support servic. **Table S3.** Multinomial logistic regression of rural-urban differences on needs of parenting support services.

## Data Availability

The datasets used and/or analyzed during the current study are available from the corresponding author on reasonable request.
