# Supplementary material for: Parenting and parenting resources among Chinese parents with children under three years of age: rural and urban differences
Source: BMC Prim Care. 2023 Feb 1;24:38. doi: 10.1186/s12875-023-01993-y (PMC9890422; doi:10.1186/s12875-023-01993-y)
Supplement: Supplementary file 1 — Additional file 1: Table S1. General linear regression of rural-urban differences in parenting, family adjustment, and parenting efficacy. Table S2. Binary logistic regression of rural-urban differences on utilization of parenting support servic. Table S3. Multinomial logistic regression of rural-urban differences on needs of parenting support services. [file 12875_2023_1993_MOESM1_ESM.docx]

Table S1. General linear regression of rural-urban differences in parenting, family adjustment, and parenting efficacy

|  | Parenting  *β (SE)* | | | |  | Family adjustment  *β (SE)* | | |  | Parenting efficacy  *β (SE)* |
| --- | --- | --- | --- | --- | --- | --- | --- | --- | --- | --- |
|  | Parental consistency | Coercive parenting | Positive encouragement | Parent-child relationship |  | Parental adjustment | Family relationships | Parental teamwork |  |  |
| Residency |  |  |  |  |  |  |  |  |  |  |
| Urban | ref | ref | ref | ref |  | ref | ref | ref |  | ref |
| Rural | 0.14 (0.10) | 0.32 (0.10) ** | -0.22 (0.11) * | -0.31 (0.10) ** |  | 0.21 (0.06) ** | 0.18 (0.06) ** | 0.07 (0.07) |  | -0.15 (0.19) |
| Parents’ age (years) | -0.01 (0.01) | -0.03 (0.01) ** | -0.02 (0.01) | -0.002 (0.01) |  | -0.01 (0.01) | -0.01 (0.01) | -0.0003 (0.01) |  | 0.02 (0.02) |
| Monthly income per capita (CNY/thousands) | -0.01 (0.01) | -0.01 (0.01) | 0.02 (0.01) | 0.02 (0.01) |  | -0.01 (0.01) | -0.01 (0.01) | -0.01 (0.01) |  | 0.03 (0.02) |
| Marital status |  |  |  |  |  |  |  |  |  |  |
| Other | ref | ref | ref | ref |  | ref | ref | ref |  | ref |
| Married | -0.03 (0.26) | -0.04 (0.25) | 0.45 (0.29) | 0.33 (0.25) |  | -0.48 (0.18) ** | -0.76 (0.19) ** | -0.35 (0.65) ** |  | 0.83 (0.56) |
| Number of children |  |  |  |  |  |  |  |  |  |  |
| >=3 | ref | ref | Ref | ref |  | ref | ref | ref |  | ref |
| 2 | 0.28 (0.34) | 0.39 (0.33) | 1.02 (0.39) ** | 0.50 (0.36) |  | -0.23 (0.21) | -0.30 (0.21) | -0.59 (0.25) * |  | -0.004 (0.65) |
| 1 | 0.26 (0.34)  0.449 | 0.19 (0.33)  0.566 | 1.05 (0.39)  0.007 | 0.51 (0.36)  0.161 |  | -0.22 (0.21)  0.283 | -0.29 (0.22)  0.186 | -0.65 (0.25)  0.009 |  | 0.21 (0.66)  0.745 |
| Age of the target child (months) | -0.01 (0.01) | 0.01 (0.01) | 0.002 (0.01) | 0.002 (0.01) |  | 0.003 (0.002) | 0.01 (0.002) * | 0.01 (0.003) ** |  | 0.01 (0.01) |
| Other caregivers caring for the child |  |  |  |  |  |  |  |  |  |  |
| yes | ref | ref | ref | ref |  | ref | ref | ref |  | ref |
| no | -0.03 (0.17) | -0.03 (0.16) | 0.19 (0.19) | 0.06 (0.16) |  | -0.11 (0.09) | -0.17 (0.10) | -0.06 (0.10) |  | 0.13 (0.28) |

Notes. ref, reference level. **p*＜0.05，***p*＜0.01

Table S2. Binary logistic regression of rural-urban differences on utilization of parenting support services

|  | *β (SE)* | OR | *95%CI* | *p* |
| --- | --- | --- | --- | --- |
| Residency |  |  |  |  |
| Urban | ref | ref | ref | ref |
| Rural | -0.80 (0.49) | 0.45 | (0.17, 1.16) | 0.099 |
| Parents’ age (years) | -0.02 (0.05) | 0.99 | (0.89, 1.10) | 0.774 |
| Monthly income per capita (CNY/thousands) | 0.01 (0.06) | 1.01 | (0.90, 1.13) | 0.099 |
| Marital status |  |  |  |  |
| Other | ref | ref | ref | ref |
| Married | 0.53 (1.01) | 1.69 | (0.24, 12.18) | 0.602 |
| Number of children |  |  |  |  |
| >=3 | ref | ref | ref | ref |
| 2 | 0.86 (1.51) | 2.35 | (0.12, 45.31) | 0.571 |
| 1 | 0.77 (1.51) | 2.15 | (0.11, 41.31) | 0.611 |
| Age of the target child (months) | 0.002 (0.02) | 1.00 | (0.97, 1.04) | 0.908 |
| Other caregivers caring for the child |  |  |  |  |
| yes | ref | ref | ref | ref |
| no | 0.17 (0.72) | 1.18 | (0.29, 4.81) | 0.816 |

Notes. The dependent variable utilization was coded as 0=Yes and 1=No. ref, reference level.

Table S3. Multinomial logistic regression of rural-urban differences on needs of parenting support services

|  | No Needs | | | |  | Unsure Needs | | | |
| --- | --- | --- | --- | --- | --- | --- | --- | --- | --- |
|  | *β (SE)* | OR | *95%CI* | *p* |  | *β (SE)* | OR | *95%CI* | *p* |
| Residency |  |  |  |  |  |  |  |  |  |
| Urban | ref | ref | ref | ref |  | ref | ref | ref | ref |
| Rural | 0.19 (0.47) | 1.20 | (0.48, 3.03) | 0.777 |  | -0.12 (0.35) | 0.89 | (0.45, 1.76) | 0.728 |
| Parents’ age (years) | -0.13 (0.07) | 0.88 | (0.77, 1.00) | 0.045 |  | -0.15 (0.05) | 0.86 | (0.78, 0.94) | 0.001 |
| Monthly income per capita (CNY/thousands) | -0.07 (0.08) | 0.93 | (0.80, 1.10) | 0.385 |  | 0.05 (0.04) | 1.05 | (0.98, 1.13) | 0.179 |
| Marital status |  |  |  |  |  |  |  |  |  |
| Other | ref | ref | ref | ref |  | ref | ref | ref | ref |
| Married | -0.51 (0.98) | 0.60 | (0.08, 4.08) | 0.602 |  | 1.06 (1.15) | 2.87 | (0.30, 27.26) | 0.358 |
| Number of children |  |  |  |  |  |  |  |  |  |
| >=3 | ref | ref | ref | ref |  | ref | ref | ref | ref |
| 2 | -1.81 (0.97) | 0.16 | (0.03, 1.09) | 0.061 |  | -0.69(1.17) | 0.50 | (0.05, 4.95) | 0.555 |
| 1 | -2.23 (1.00) | 0.11 | (0.02, 0.76) | 0.026 |  | -1.21(1.18) | 0.30 | (0.03, 2.98) | 0.297 |
| Age of the target child (months) | 0.02 (0.02) | 1.02 | (0.98, 1.05) | 0.334 |  | 0.01 (0.01) | 1.01 | (0.99, 1.04) | 0.266 |
| Other caregivers caring for the child |  |  |  |  |  |  |  |  |  |
| yes | ref | ref | ref | ref |  | ref | ref | ref | ref |
| no | 0.28 (0.82) | 1.32 | (0.27, 6.56) | 0.733 |  | -0.004 (0.50) | 1.00 | (0.38,2.63) | 0.993 |

Notes. The dependent variable Needs of Parenting Support Serivce was coded as 0=yes (reference level), 1=No needs, and 2=Unsure needs. ref, reference level.
